# Activation of the kynurenine pathway and increased production of the excitotoxin quinolinic acid following traumatic brain injury in humans

**DOI:** 10.1186/s12974-015-0328-2

**Published:** 2015-05-30

**Authors:** Edwin B. Yan, Tony Frugier, Chai K. Lim, Benjamin Heng, Gayathri Sundaram, May Tan, Jeffrey V. Rosenfeld, David W. Walker, Gilles J. Guillemin, Maria Cristina Morganti-Kossmann

**Affiliations:** Department of Physiology, Monash University, Clayton, VIC 3800 Australia; Department of Pharmacology and Therapeutics, The University of Melbourne, Melbourne, Australia; Neuroinflammation group, Faculty of Medicine and Health Sciences, Macquarie University, Sydney, Australia; Applied Neurosciences Program, Peter Duncan Neurosciences Research Unit, St Vincent’s Centre for Applied Medical Research, Sydney, Australia; Hospital Queen Elizabeth, Karung Berkunci No. 2029, 88586 Kota Kinabalu, Sabah Malaysia; Department of Neurosurgery, The Alfred Hospital, Melbourne, Australia; Department of Surgery, Central Clinical School and Monash Institute of Medical Engineering, Monash University, Melbourne, Australia; The Ritchie Centre, Hudson Institute of Medical Research, Monash Medical Centre, Melbourne, Australia; Australian New Zealand Intensive Care Research Centre, Department of Epidemiology and Preventive Medicine, Monash University, Melbourne, Australia; Department of Child Health, Barrow Neurological Institute, University of Arizona, Phoenix, AZ USA

**Keywords:** Patients, Traumatic brain injury, Kynurenine pathway, Tryptophan metabolism, Quinolinic acid

## Abstract

**Abstract:**

During inflammation, the kynurenine pathway (KP) metabolises the essential amino acid tryptophan (TRP) potentially contributing to excitotoxicity via the release of quinolinic acid (QUIN) and 3-hydroxykynurenine (3HK). Despite the importance of excitotoxicity in the development of secondary brain damage, investigations on the KP in TBI are scarce. In this study, we comprehensively characterised changes in KP activation by measuring numerous metabolites in cerebrospinal fluid (CSF) from TBI patients and assessing the expression of key KP enzymes in brain tissue from TBI victims. Acute QUIN levels were further correlated with outcome scores to explore its prognostic value in TBI recovery.

**Methods:**

Twenty-eight patients with severe TBI (GCS ≤ 8, three patients had initial GCS = 9–10, but rapidly deteriorated to ≤8) were recruited. CSF was collected from admission to day 5 post-injury. TRP, kynurenine (KYN), kynurenic acid (KYNA), QUIN, anthranilic acid (AA) and 3-hydroxyanthranilic acid (3HAA) were measured in CSF. The Glasgow Outcome Scale Extended (GOSE) score was assessed at 6 months post-TBI. Post-mortem brains were obtained from the Australian Neurotrauma Tissue and Fluid Bank and used in qPCR for quantitating expression of KP enzymes (indoleamine 2,3-dioxygenase-1 (IDO1), kynurenase (KYNase), kynurenine amino transferase-II (KAT-II), kynurenine 3-monooxygenase (KMO), 3-hydroxyanthranilic acid oxygenase (3HAO) and quinolinic acid phosphoribosyl transferase (QPRTase) and IDO1 immunohistochemistry.

**Results:**

In CSF, KYN, KYNA and QUIN were elevated whereas TRP, AA and 3HAA remained unchanged. The ratios of QUIN:KYN, QUIN:KYNA, KYNA:KYN and 3HAA:AA revealed that QUIN levels were significantly higher than KYN and KYNA, supporting increased neurotoxicity. Amplified IDO1 and KYNase mRNA expression was demonstrated on post-mortem brains, and enhanced IDO1 protein coincided with overt tissue damage. QUIN levels in CSF were significantly higher in patients with unfavourable outcome and inversely correlated with GOSE scores.

**Conclusion:**

TBI induced a striking activation of the KP pathway with sustained increase of QUIN. The exceeding production of QUIN together with increased IDO1 activation and mRNA expression in brain-injured areas suggests that TBI selectively induces a robust stimulation of the neurotoxic branch of the KP pathway. QUIN’s detrimental roles are supported by its association to adverse outcome potentially becoming an early prognostic factor post-TBI.

## Introduction

Traumatic brain injury (TBI) is one of the leading causes of morbidity and mortality in healthy individuals. Epidemiologic studies reported that TBI occurs more frequently in young adults due to motor vehicle accidents, sport activities and assaults as major causes of injury. Although advanced progress in pre-hospital intervention, neuroimaging, emergency management and intensive care as well as neurosurgical techniques have reduced mortality after severe TBI, the overall outcome remains poor with patients suffering from long-term disability and mental illness.

TBI is distinguished in two phases, namely primary and secondary injury. Primary injury occurs at the time of trauma resulting from either direct physical impact (focal trauma) or inertial force induced by rapid acceleration-deceleration (diffuse trauma) or a combination of both [1]. Subsequently, long-lasting secondary injury processes commence lasting for minutes, hours and days following a TBI. The combination of such pathological changes in the brain’s intrinsic physiology and biochemistry aggravates brain damage and possibly underlies the causes of death. Over the past decades, compelling evidence has shown that such delayed processes represent the most destructive phase of TBI; however, the complexity and the mechanisms of secondary brain injury require further elucidation. There are few well-defined secondary pathways including inflammation, oxidative stress and excitotoxicity that terminate with the release of neurotoxins, leading to delayed cell death.

The study presented in this paper aimed to investigate changes in cerebral activation of the kynurenine pathway (KP) induced by severe TBI, with a specific focus on the potential roles of the terminal end product of the KP, the neurotoxin quinolinic acid (QUIN) [2] in predicting patients’ long-term outcome.

The KP is the major cascade for metabolism of the essential amino acid tryptophan (TRP). TRP is the only amino acid bound to albumin to form a TRP-albumin complex in the blood stream [3]. This complex serves as a buffering system for maintaining a relative constant level of free TRP in blood. Free circulating TRP can be transported across the blood-brain barrier into the brain by the sodium-independent amino acid transporter system [4]. TRP is required for the synthesis of all proteins to ensure cell survival in both the periphery and central nervous system. It also strongly influences the function of the immune system in that reduction in TRP concentration suppresses the proliferation of peripheral mononuclear cells [5], decreases the activation of allogenic immune cells [6] and enhances the inhibition of T-cell responses [7]. In this regard, *in vivo* studies have shown that blocking the activity of the TRP-metabolising enzyme, indoleamine 2,3-dioxygenase-1 (IDO1), in the brain results in maintenance of the TRP pool and consequently the proliferation of T cells [8]. In the brain, TRP is metabolised to kynurenine (KYN) by IDO1 in neurons, astrocytes, microglia and infiltrated macrophages. KYN is then further metabolised into other neuroactive products of the KP such as kynurenic acid (KYNA), anthranilic acid (AA), 3-hydroxykynurenine (3HK), 3-hydroxyanthranilic acid (3HAA), QUIN, picolinic acid (PA) and nicotinic acid (NA) [9].

Numerous metabolites of the KP have received considerable attention since they display neuroactive, neurotoxic or immunomodulatory properties [10]. In fact, while QUIN acts as an excitotoxic agonist to the N-methyl-D-aspartate (NMDA) receptor, KYNA is rather considered a neuroprotectant through its antagonising action to the NMDA receptor, thus having an opposite functional role to its counterparts [11].

Therefore, understanding the changes in this complex pathway that are triggered in pathological conditions is critical for the future development of therapies to reduce secondary brain tissue damage. These KP metabolites are implicated in a variety of neuroinflammatory disorders including Alzheimer’s disease, amyotrophic lateral sclerosis (ALS) and the AIDS-dementia complex [9, 12–16]. With the pathogenesis of neurological diseases, QUIN is likely one of the most important end products of the KP (review by [17]). In the primate and human brain, QUIN levels are strongly induced by interferon gamma (IFN-γ), a cytokine that is upregulated during infection and inflammation [12, 18] and following hypoxia-ischemia [19]. QUIN displays the ability to bind and activate the NMDA receptor causing prolonged Ca^2+^ influx, with loss of intra- and extra-cellular ionic balance in neurons; it increases cell membrane permeability, contributes to vasogenic oedema and ultimately leads to cell death [17].

Animal experiments employing intrastriatal injection of QUIN resulted in substantial neuronal cell loss [20, 21], whereas peripheral QUIN administration increased cerebral astrogliosis and oxidative stress [22]. Delivery of QUIN into the brain produced a progressive mitochondrial dysfunction, impaired cellular energy homeostasis [23, 24], increased oxidative stress [25] and enhanced nitric oxide (NO) synthase activities [26]. Even a modest (~200 nM) but prolonged elevation of QUIN in the adult brain is sufficient to reduce dendritic varicosities and microtubular assemblies of human neurons *in vitro* [27].

Despite the relevance of QUIN production in the pathogenesis of many neurological conditions, there are only limited studies that reported changes in the levels of QUIN and no reports at all in the TBI setting demonstrating changes of other metabolites of the KP. Published data showed that increased concentration of QUIN in cerebrospinal fluid (CSF) is strongly associated with mortality in children and adults after TBI [28, 29]. However, these early reports have not assessed how elevated QUIN production relates to other metabolites of the KP, which presents intrinsic branches potentially leading to opposite, neuroprotective or neurotoxic sequel. Therefore, further understanding of the activation of the KP consequent to TBI is pivotal to pinpoint its roles in the progress of secondary brain damage and develop a pharmacological intervention targeting the specific step of this complex pathway responsible for excessive QUIN synthesis [30].

In this study, we embarked on a comprehensive characterisation of the KP by measuring six metabolites in CSF collected from patients with severe TBI. In order to elucidate the relationship between these metabolites and the activation state of the KP, the level of expression of six enzymes of the KP was examined using post-mortem brain tissue obtained from individuals who died of TBI. This study design hinges on the hypothesis that TBI activates cerebral KP, enhances the production of neurotoxin QUIN and increases mRNA expression of enzymes regulating QUIN production in the brain. We further hypothesised that QUIN concentrations in CSF are correlative with Glasgow Outcome Scale Extended (GOSE) scores assessed at 6 months following TBI. Therefore, measuring QUIN in the clinic may aid in assessing long-term outcomes in severe TBI patients.

## Materials and methods

### Patient recruitment and CSF and serum sample collection

The study was conducted in accordance with the National Statement on Ethical Conduct in Research Involving Humans of the National Health and Medical Research Council of Australia and was approved by the Human Ethics Committee of the Alfred Hospital, Melbourne, Australia. Twenty-eight severe TBI patients were recruited from the Trauma Service of the Alfred Hospital (Table 1). Formal consent was obtained from the next of kin before the commencement of the study. The patient’s inclusion criteria included severe TBI, established by a post-resuscitation, pre-intubation Glasgow Coma Scale (GCS) ≤ 8 (three patients had initial GCS = 9–10 but rapidly deteriorated requiring intubation at the scene) and implantation of an extraventricular drain (EVD) for monitoring and decreasing intracranial pressure via drainage of CSF. Exclusion criteria included age below 18 years, pregnancy, history of neurodegenerative disease, infectious disease, previous TBI and cancer.

**Table 1:** Demographic information of TBI patients

Variables	Values
Age, years, median (range)	35 (21–69)
Gender, *n* (%)	
Males	22 (62.9)
Females	6 (17.1)
Type of accident, *n* (%)	
Motor vehicle	14 (50.0)
Motorbicycle	5 (17.9)
Pedestrian	4 (14.3)
Fall	3 (10.7)
Other	2 (7.1)
GCS, median (range)	5.5 (3–10)
GCS ≤ 8, n (%)	25 (89.3)
ISS, median (range)	36.5 (13–57)
GOSE, median (range)	4 (1–8)
Unfavourable outcome (1–4), *n* (%)	18 (64.3)
GOSE 1	6
GOSE 2	3
GOSE 3	4
GOSE 4	5
Favourable outcome (5–8), *n* (%)	10 (35.7)
GOSE 5	4
GOSE 6	3
GOSE 7	1
GOSE 8	2

A total of 28 patients with severe TBI were recruited in the study for longitudinal collection of CSF and serum. The following clinical parameters were recorded: Glasgow Coma Scale (GCS): severe ≤8. Injury Severity Score (ISS): 0 = no injury, 75 = maximal untreatable injury. Glasgow Outcome Scale Extended (GOSE): 1 = dead, 2 = vegetative state, 3 = lower severe disability, 4 = upper severe disability, 5 = lower moderate disability, 6 = upper moderate disability, 7 = lower good recovery, 8 = upper good recovery

Management of TBI patients included preliminary CT scans within 4 h of admission to assess the extent of injury and surgical implantation of an *in situ* intracranial pressure (ICP) probe coupled with an EVD. CSF was drained when the ICP was greater than 20 mmHg and accumulated in a drainage bag over 24 h. The CSF was collected daily by ICU research staff for six consecutive days from admission to the hospital (day 0) until day 5 post-TBI. CSF was centrifuged at 2000×*g* for 15 min at 4 °C, and the supernatant was stored at −80 °C until analysis. Blood samples were also collected daily and centrifuged, and serum was stored at −80 °C.

### Assessment of outcomes

The outcome was assessed using the Glasgow Outcome Scale Extended (GOSE) at 6 months post-injury. Phone interviews with individual patients, their family members or other informants were conducted by experienced research nurses using the questionnaire of Wilson et al. [31]. The GOSE is an examiner-dependent assessment; therefore, research staffs conducting the interviews are extensively trained to produce high-level consistency among patients. GOSE aims to gauge the patient’s social, occupational, behavioural and neurological recovery after TBI, where GOSE 1 = death, 2 = vegetative state, 3 = severe disability (lower band), 4 = severe disability (upper band), 5 = moderate disability (lower band), 6 = moderate disability (upper band), 7 = good recovery (lower band) and 8 = good recovery (upper band).

### Control patients and sample collection

Control CSF was obtained from patients undergoing elective neurosurgery for implantation of ventriculo-peritoneal shunts following a diagnosis of hydrocephalus (*n* = 11, 6 males and 5 females, between the ages of 30 and 74 years). Ethics approval was granted by the Alfred Human Ethics Board and informed consent was obtained prior surgery. Exclusion criteria included involvement of neurodegenerative diseases, cancer, infectious disease, intracerebral haemorrhages or previous TBI. Control serum samples (*n* = 20) were obtained from 12 female and 8 male healthy volunteers between the ages of 21 and 55 years. Both serum and CSF samples were processed using the same methods as described for TBI patients.

### Measurement of tryptophan metabolites in CSF and serum

CSF samples were analysed by high-performance liquid chromatography (HPLC) and gas chromatography-mass spectrometry (GC-MS) methods to quantify the concentration of the metabolites of the KP, namely TRP, KYN, KYNA, AA, 3HAA and QUIN. TRP was only measured in serum. Proteins in CSF and serum were first precipitated by adding equal amount of 5 % trichloroacetic acid (*w*/*v*, Sigma-Aldrich, St. Louis, MO, USA) to the sample and then centrifuged for 10 min at 2000×*g* at 4 °C. The supernatants were filtered through 0.2-μm filters (Waters, Rydalmer, NSW, Australia) before use in HPLC.

The HPLC methods for quantification of TRP, KYN, KYNA, AA and 3HAA were based on Darlington et al. [32] with slight modification. The mobile phase of the TRP, KYN and KYNA assay consisted of 50 mM sodium acetate (Merck, Whitehouse Station, NJ, USA), 250 mM zinc acetate (Sigma-Aldrich, St. Louis, MO, USA) and 5 % acetonitrile (Merck, Whitehouse Station, NJ, USA). The mobile phase was buffered to pH 5.5 by glacial acetate acid (Merck, Whitehouse Station, NJ, USA), filtered through a 0.45-μm filter and pumped at a flow rate of 1 ml/min. The mobile phase of AA and 3HAA consisted of 25 mM sodium acetate and 2.5 % acetonitrile, buffered at pH 5.5, filtered and pumped at a flow rate of 1 ml/min. Stock standards of TRP, KYN, KYNA, AA and 3HAA were made in concentration of 250 mM using high-purity chemical from Sigma-Aldrich (St. Louis, MO, USA). The stock standard solution was kept at 4 °C for 1 month. Working standards were made by further dilution of the stock solution to establish standard curves of 1.25, 2.5, 6.25 and 12.5 μM for TRP and KYN; 10, 20, 60, 120 and 200 nM for KYNA; and 5, 15, 30 and 50 pM for AA and 3HAA. Quality control samples were prepared for each metabolite, aliquoted in multiple vials and stored at −80 °C. Deproteinised human CSF and serum samples (100 μl), standards and quality controls were injected into HPLC. The retention time and peak height of each metabolite from each sample were quantified using either WinChrom Chromatography Data System (GBC, Melbourne, Australia) or Waters Millennium Chromatography Manager (Waters, Rydalmer, NSW, Australia). TRP, KYN and KYNA were separated using a polymeric column (20 × 3.2 mm; 5-μm particle size; Agilent Technologies, Forest Hill, VIC, Australia) and detected by an absorbance detector for TRP and KYN (Photodiode Array Detector, Shimadzu SPD-M10A (Kyoto, Japan); absorbance wavelength TRP = 278 nm; KYN = 363 nm) or a fluorescent detector for KYNA (Waters 474 Scanning Fluorescence Detector, excitation = 344 nm, emission = 388 nm) connected in series. AA and 3HAA were measured by a Synergi Hydro column (250 × 4.60 mm; 4 μm particle size; Phenomenex (Torrance, CA, USA)) connected to a fluorescent detector (Waters 474 Scanning Fluorescence Detector, Ex = 320 nm, Em = 420 nm). For each set of sample measurement, fresh working standards were prepared and measured together with unknown samples and quality controls. A standard curve was plotted using linear regression of the area under the chromatograph of the corresponding concentration of the standards. The concentrations of TRP, KYN, KYNA, AA and 3HAA were calculated against the standard curves from the corresponding run of the assay. Quality controls were used to calculate inter- and intra-assay coefficient.

QUIN in CSF was measured by GC-MS as described by Smythe et al. [33]. Briefly, a standard curve of QUIN (10, 20, 50, 100, 200 and 500 nM) was prepared from a stock solution (2 μM). An internal standard containing 50–200 fmol of [^2^H_3_]-QUIN in 50 μl was added into 100-μl deproteinised samples. These samples were then dried by N_2_ stream. The residues were mixed with trifluoroacetic anhydride (100 μl, Sigma-Aldrich, St. Louis, MO, USA) and hexafluoroisopropanol (100 μl; Sigma-Aldrich, St. Louis, MO, USA) and left at room temperature overnight to produce the dihexafluoroisopropyl ester from QUIN. The dihexafluoroisopropyl ester product was dissolved in toluene (1 ml, Sigma-Aldrich, St. Louis, MO, USA), washed with 5 % NaHCO_3_ (1 ml; Sigma-Aldrich, St. Louis, MO, USA) and ddH_2_O (1 ml) and dried over anhydrous sodium sulphate (~500 mg; Sigma-Aldrich, St. Louis, MO, USA). The samples were then transferred into an auto-sampler (7683, Agilent Technologies, Forest Hill, VIC, Australia), and 1 μl was injected into a HP-5MS capillary column (30 m × 0.25 mm; Agilent Technologies, Forest Hill, VIC, Australia) connected to an Agilent 6890 gas chromatograph and an Agilent mass selective detector (Agilent Technologies, Forest Hill, VIC, Australia).

### Post-mortem brain tissue collection

All procedures were conducted in accordance with the Australian National Health & Medical Research Council’s National Statement on Ethical Conduct in Human Research (2007), the Victorian Human Tissue Act 1982, the National Code of Ethical Autopsy Practice and the Victorian Government Policies and Practices in Relation to Post-Mortem.

Trauma brain samples from 16 individuals who died after closed head injury were obtained from the Australian Neurotrauma Tissue and Fluid Bank. Cases were aged between 18 and 78 years (mean 49.9 years), and the causes of injury include motor vehicle accident, motorbike accident, nursing home accident, household accident, stair accident and falls (see Table 2 for clinical information and epidemiological details). The post-mortem intervals varied between 40 and 129 h (mean 80.6 h).

**Table 2:** Details of the 16 trauma and 10 control cases

Case	Age (years)	Sex	Cause of injury	PMI (h)	Cause of death	Survival time
1	51.1	M	Motor vehicle accident	60	Brain + multiple injuries	<17 min
2	78.7	M	Nursing home accident	45	Brain injury	<17 min
3	27	M	Suicide	84	Brain + multiple injuries	<17 min
4	18.3	M	Motor vehicle accident	79	Brain + multiple injuries	<17 min
5	57.9	F	Motor vehicle accident	87	Brain + multiple injuries	<17 min
6	49	M	Motor vehicle accident	107	Brain + multiple injuries	<17 min
7	34.7	M	Motorbike accident	66	Brain + multiple injuries	<17 min
8	21.5	M	Motor vehicle accident	100	Brain injury	<17 min
9	57.6	F	Motor vehicle accident	97	Brain injury	<17 min
10	46.0	M	Fall	129	Brain injury	6 h
11	56.3	M	Motor vehicle accident	65	Brain injury	8 h
12	64.6	M	Fall	61	Brain injury	8 h
13	75.9	M	Staircase fall	89	Brain injury	10 h
14	59.6	F	Motor vehicle accident	80	Brain injury	35 h
15	61.7	M	Fall	40	Brain injury	93 h
16	38.9	F	Staircase fall	101	Brain injury	122 h
17	16	M	-	-	Suicide by hanging	-
18	48.7	M	-	50	Cardiac failure	-
19	51.6	M	-	64	Asthma	-
20	52.3	M	-	52	Cardiomyopathy	-
21	59.6	M	-	43	Pulmonary embolism	-
22	64.1	M	-	24	Ischaemic heart disease	-
23	66.9	M	-	10	Pneumonia	-
24	64.4	M	-	24	Pulmonary embolism	-
25	77.5	M	-	53	Myocardial infarction	-
26	60	F	-	48	Myocardial infarction	-

Cases 1–9: cases with a survival time between 0 and 17 min; cases 10–16: cases with a survival time between 6 and 261 h; cases 17–26: control cases. All brains were obtained at autopsy

*PMI* post-mortem interval (time between death and brain retrieval), *M* male, *F* female

Tissues were divided into four groups—Control, Acute Death, Delayed Death Injured Tissue and Delayed Death Normal Tissue. The Acute Death group includes nine patients (seven males and two females) who were pronounced dead upon the arrival of paramedics at the scene of accident (survival time <17 min). In those victims with an absence of visible tissue damage at post-mortem, cortical tissue was collected directly under the area having marked injury to the skull and skin. The group of delayed death includes seven patients (five males and two females) who survived more than 6 h post-TBI (range of 6–122 h, mean survival time of 40 h). At autopsy, two full coronal brain slices were taken by a forensic pathologist at 1 cm posterior to the mammillary bodies at the level of the basal ganglia. One slice was fresh frozen for PCR experiments, and the other was fixed in formalin for immunohistochemistry. The tissue used in these experiments was taken from the cortical region of these coronal slices in areas showing visible tissue damage (Delayed Death Injured Tissue) and from a corresponding region on the contralateral side of the brain without macroscopic injury (Delayed Death Normal Tissue). These groupings were consistent with our previous reports [34–36].

For comparison, the same regions analysed in TBI tissues were obtained from control brain samples. These were provided by the National Neural Tissue Resource Centre of Australia (*n* = 13). These brains originated from individuals with no history of TBI or neuropathology; the age range matched that of TBI population and was between 16 and 78 years old (mean 58 years old). A neuropathologist, Prof. C. McLean, Head of the Department of Anatomical Pathology at the Alfred Hospital, performed brain tissue pathology and injury identification.

### Gene expression of enzymes regulating the kynurenine pathway

The RNA isolation and cDNA preparation was performed using a well-characterised methodology as described previously in these brains by our group [36]. Briefly, TRIzol Plus RNA purification kit was used for RNA extraction from fresh frozen cortex tissue. A Nanodrop1000 spectrophotometer (Thermo Fisher Scientific, Wilmington, DE, USA) was used to determine the concentration and purity of the RNA samples. A minimum ratio of absorbance at 260 and 280 nm of 2 was considered as pure RNA. Agilent 2100 bioanalyzer (Agilent Technologies, Waldbronn, Germany) was used to assess the integrity of extracted RNA. RNA quality is considered good if the sample has a minimum RNA integrity number (RIN) value of 6. Oligo d(T)20 was then used as primers to reverse transcribed RNA fraction into cDNA using SuperScript III reverse transcriptase according to the manufacturer’s protocol (Invitrogen, Carlsbad, CA, USA). Quantitative PCR was performed using TaqMan primer for kynurenase (KYNase, Hs00187560_m1), kynurenine amino transferase-II (KAT-II, Hs00212039_m1), kynurenine 3-monooxygenase (KMO, Hs00175738_m1), 3-hydroxyanthranilic acid oxygenase (3HAO, Hs00201915_m1) and quinolinic acid phosphoribosyl transferase (QPRTase, Hs00204757_m1) (Applied Biosystems, Foster City, CA, USA). Hydroxymethylbilane synthase (HMBS, Hs00609297_m1), peptidylpropyl isomerase A (cyclophilin A) (PPIA, Hs99999904_m1), ubiquitin C (UBC, Hs00824723_m1) and glyceraldehyde-3-phosphate dehydrogenase (GAPDH, Hs99999905_m1) (Applied Biosystems, Foster City, CA, USA) have the most stable mRNA levels in post-mortem brain tissue and were used as endogenous control.

Quantitative PCR (qPCR) reaction mixture for each sample contains 1 μl of cDNA, 12.5 μl Taq-Man Universal PCR Master Mix (Applied Biosystems, Foster City, CA, USA), 1.25 μl of primer and 10.25 μl nuclease-free H_2_O. Seven microlitres of the reaction mixture was added into a 384 well plate, and qPCRs were performed using the 7900HT Fast Real-Time PCR system (Applied Biosystems, Foster City, CA, USA). All experiments were performed in triplicate. The level of mRNA expression of KP enzymes was quantified using the comparative Ct method (∆∆Ct). This method used an arithmetic formula (2^−∆∆Ct^) instead of a standard curve for the calculation of the relative KP enzyme expression level. This method can be used in place of the standard curve as long as both target and housekeeping genes have been validated to have approximate equal efficiencies. The mRNA levels of KP enzymes were normalised using the averaged level of endogenous control gene (HMBS, PPIA, UBC and GAPDH).

For IDO1, the primer forward (GCCAGCTTCGAGAAAGAGTTG) and reverse (TGACTTGTGGTCTGTGAGATGA) were made based on Harvard primer bank (http://pga.mgh.harvard.edu/primerbank/, assessed on April 2012). qPCR reactions were performed in a final volume of 10 μl. Each reaction mixture contains 5 μl Fast SYBR® green master mix, 5 μM forward and reverse primers and 125 ng of cDNA template. The reaction was incubated at 95 °C for 20 s and amplified for 40 cycles of 95 °C for 1 s and 55 °C for 20 s.

### IDO1 immunohistochemistry

Localisation and distribution of cells expressing IDO1 were identified using immunohistochemical staining. Cortical tissues were immersion fixed in 4 % paraformaldehyde for 72 h before paraffin-embedding process. Immunohistochemistry was performed on 7-μm sections using a Dako Autostainer XL (DakoCytomation, Carpinteria, CA, USA). In brief, sections were deparaffinised and rehydrated and then subjected to antigen retrieval in a citrate buffer (Dako (Cambridge, UK), S2367, pH 9) within a pressurised heating chamber (Dako (Cambridge, UK), S2800, 125 °C, 20 psi) for 2 min. Quenching of endogenous peroxidase activity was achieved using 3 % H_2_O_2_. Sections were then treated with serum-free protein blocker (Dako (Cambridge, UK), X0909) before 120-min incubation with antibody against IDO1 (IDO-MCA, 1:100, Oriental Yeast Co., Ltd., Tokyo, Japan). Envision HRP-linked polymer (Dako (Cambridge, UK), K4001) and 3,3-diaminobenzidine (Dako (Cambridge, UK), K3468) were applied to visualise positive staining. Tissue sections were counterstained with haematoxylin on a Leica Autostainer XL (North Ryde, NSW, Australia) and coverslipped using a Leica CV5030 device (North Ryde, NSW, Australia). Isotype-matched monoclonal antibody was used as a negative control. All sections were examined under a light microscope (Leica DM2500, North Ryde, NSW, Australia) and positive-stained cells were counted semi-quantitatively by ImageJ software as number of positive cell × 100/total number of cells in ten random microscopic (×200) fields in each section. Statistical analyses were performed using GraphPad Prism Version 6 software (mean ± SE). All tests were two-tailed with 95 % confidence interval values (*p* < 0.05).

### Statistical analysis

#### Tryptophan metabolites

Statistical analyses were undertaken using GraphPad Prism 5. Generally, for all data sets, we first tested the data for homogeneity of variance using Bartlett’s test prior to applying further analysis. Due to non-normally distributed metabolite data, logarithmic transformation was applied before analysis using one-way ANOVA and Dunnett *post hoc* for multiple comparisons between control group and each day after TBI. Differences in QUIN levels in CSF between favourable (GOSE 5–8) and unfavourable outcome (GOSE 1–4) was determined by Student’s *t*-test following logarithmic transformation. To reflect logarithmic transformation of data, results were presented as geometric means with 95 % confidence intervals throughout. Correlation between QUIN and GOSE was conducted using Spearman’s correlation coefficient. A *p* value of less than 0.05 was considered as statistically significant.

#### qPCR

Statistical analyses were undertaken using GraphPad Prism 5. Due to non-normally distributed data; logarithmic transformation was applied before analysed using 1-way ANOVA and Dunnett *post hoc* for multiple comparisons between each group. A *p* value of less than 0.05 was considered as statistically significant.

## Results

### Patient demographics and outcome

A total of 28 TBI patients were recruited immediately after resuscitation at the accident scene and admitted to the ICU. TBI patients’ age ranged between 21 and 69 years (median age of 35 years) with a majority of males (*n* = 22, 62.9 %) (Table 1). Most patients (*n* = 23) were victims of road traffic accidents (82.2 %), and the remaining patients sustained a fall (*n* = 3) or other injurious mechanisms (*n* = 2). The GCS at the scene was ≤8 for all patients with the exception of three patients who had an initial GCS of 9–10 and rapidly deteriorated requiring intubation prior to admission to the hospital. The Injury Severity Scores (ISS) were recorded to assess the combined injuries sustained including brain and peripheral trauma. Median ISS for this cohort was 36.5 ranging between 13 and 57, which is indicative of a high incidence of additional injuries to TBI. Outcome assessed at 6 months post-TBI was generally poor with a median GOSE of 4. Following dichotomisation, 18 patients had an unfavourable outcome with GOSE 1–4 (64.3 %), whereas 10 patients had a favourable outcome with GOSE 5–8 (35.7 %); 6 patients died (GOSE 1) during the first 6 months from injury.

### Changes in tryptophan metabolites and increases QUIN in CSF after TBI

**Fig. 1:**
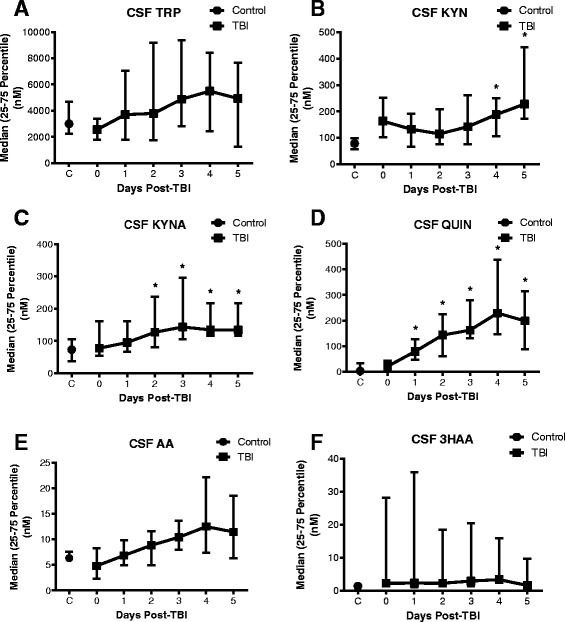
Profile of the kynurenine pathway metabolites in the CSF of patients with TBI. The concentration of six tryptophan metabolites was measured in the CSF of TBI patients consecutively from the day of hospital admission (day 0) to day 5 after injury and compared to the CSF of control individuals. **a** Tryptophan (TRP), **b** kynurenine (KYN), **c** kynurenic acid (KYNA), **d** quinolinic acid (QUIN), **e** anthranilic acid (AA) and **f** 3-hydroxyanthranilic acid (3HAA). Data is presented as daily median with 25–75 percentile. *Asterisks* indicate significant differences (*p* < 0.05) to control using one-way ANOVA followed by Dunnett’s multiple comparison as *post hoc*. TBI patients: *n* = 25–28 per time point; control group: *n* = 9–11. Significant increase of KYNA concentration in CSF were observed between days 2 and 5 when compared to the control level, while QUIN concentration was significantly increased between days 1 and 5 as to the controls

The levels of TRP in CSF showed an apparent increase between day 0 and 5 after TBI; however, the median concentrations (average median 4255 nM, 25–75 percentile: 2088–7358 nM) were not significantly different from controls (median: 3000 nM, 25–75 %: 2260–4690 nM) (Fig. 1a). In the TBI cohort, KYN displayed median levels ranging between 115.1 and 163.6 nM over the days 0 to 3 and were similar to control with a median of 78.48 nM (25–75 %: 56.21–98.62 nM; Fig. 1b). However, later in the study at days 4 and 5 post-injury, KYN concentrations became significantly elevated of at least twofold over controls with median values of 188.0 nM (25–75 %: 105.5–249.3 nM) and 228.0 nM (25–75 %: 173.1–444.1 nM; *p* < 0.05) on each day, respectively. In CSF, TBI induced an early and gradual elevation of the NMDA antagonist KYNA, which became significantly higher than controls from day 2 onwards (control median: 73.08 nM, 25–75 %: 37.86–105.8 nM; day 2 median: 127.3 nM, 25–75 %: 80.13–237.1 nM, 1.7-fold increase; *p* < 0.05; Fig. 1c). After day 2, KYNA reached a plateau that lasted until day 5 with median concentrations between 127.3 and 143.5 nM.

In contrast to KYNA, the other potent NMDA receptor agonist, QUIN, increased over control as early as day 1 to further augment from day 2 to day 4 before showing a mild decrease on day 5, which was still significantly higher than control (*p* < 0.05; Fig. 1d). In more detail, the concentration of QUIN was unchanged at day 0 (median: 21.4 nM, 25–75 %: 12.9–43.5 nM) and began to increase from day 1 (median: 79.6 nM, 25–75 %: 47.32–127.6 nM) (Fig. 1d), reaching a peak at day 4 (median: 229.4 nM, 25–75 %: 146.4–438.2 nM), which represented a tenfold increase from control levels (*p* < 0.005). Of note, the concentrations of QUIN in control CSF (median: 3 nM, 25–75 %: 3–34.5 nM) matched the normal CSF levels reported by others in the literature (<50 nM) (see review [17]).

The median concentration of AA steadily increased after TBI with an apparent peak of 12.5 nM (25–75 %: 7.4–22.2 nM) detected at day 4, which consisted of an ~2-fold increase from the control level (Fig. 1e). In comparison, the downstream product of AA and precursor of QUIN, 3HAA, displayed a 2- to 5-fold increase between days 1 and 4, despite the large variations observed throughout the study (Fig. 1f). For both AA and 3HAA, there were no statistically significant differences to control at any time point examined.

### KP activity shifts towards the production of the excitotoxic metabolite QUIN

KYNA and QUIN are end products of two distinct branches of the KP and are released at different concentrations in both normal and disease conditions [37]. We then further explored the impact of TBI to drive the KP towards excitotoxicity and QUIN production, manifested through disproportional amounts of KYNA and QUIN; we calculated the ratios of each one of these metabolites with their common precursor KYN.

**Fig. 2:**
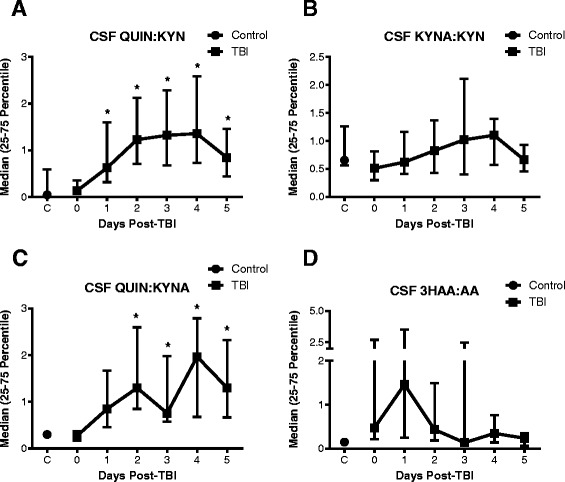
Ratios of metabolites of the kynurenine pathway indicate a higher production of quinolinic acid. The daily CSF concentrations of each metabolite were used to calculate the individual ratios depicted as follows: **a** kynurenic acid (KYNA) versus kynurenine (KYN), **b** quinolinic acid (QUIN) versus KYN, **c** QUIN versus KYNA and **d** 3-hydroxyanthranilic acid (3HAA) versus anthranilic acid (AA). Data is shown as median with 25–75 percentile. *Asterisks* indicate significant differences (*p* < 0.05) to control group using one-way ANOVA followed by Dunnett’s multiple comparison as *post hoc*. TBI patients: *n* = 25 per time point; control group: *n* = 9. Significant increase in the ratio of QUIN to KYN and QUIN to KYNA when compared to the control level indicating QUIN production increased after TBI

Interestingly, we found a significant and protracted increase in the ratio of QUIN and KYN from day 1 to day 5 post-TBI (Fig. 2a). This striking elevation reached a maximum of 28.8-fold over control levels on day 4 (*p* < 0.05). In contrast, the ratios of KYNA and KYN (range of median values from 0.51 to 1.1) as well as the ratios of 3HAA and AA (range of median values from 0.24 to 1.46) remained unchanged throughout the study and were not different from controls (median: 0.65) (Fig. 2b–d). The overproduction of QUIN was also supported by the ratio of QUIN and its neuroprotectant counterpart, KYNA, which presented significantly higher values from day 2 to day 5 after TBI (median: day 2 = 1.3, day 3 = 0.76, day 4 = 1.97 and day 5 = 1.3) when compared with controls (median: 0.3) (Fig. 2c).

These results demonstrate substantial changes in KP activity induced by TBI, whereby metabolism of TRP was shifted towards an enhanced release of neurotoxic QUIN, rather than the alternative branches terminating with the end products KYNA or AA.

### Serum concentrations of tryptophan decrease after TBI

**Fig. 3:**
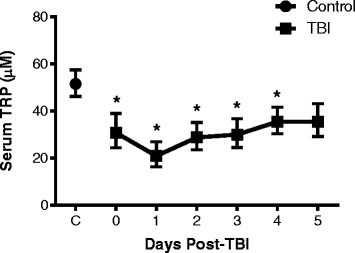
The concentration of tryptophan in serum is reduced after TBI. The concentration of tryptophan in serum samples of TBI patients was measured from the day of hospital admission (day 0) to day 5 after injury and compared to the control serum. Significant decrease in serum TRP levels were observed between days 0 and 4; there was no statistical difference at day 5 to control levels, but median concentration at day 5 (36.29 μM) was similar to day 4 (35.6 μM). Data is shown as daily median with 25–75 percentile. *Asterisks* indicate significant differences (*p* < 0.05) between TBI and control using one-way ANOVA followed by Dunnett’s multiple comparison as *post hoc*. TBI patients: *n* = 25–28 per time point; control group: *n* = 9–11

We were also interested to determine how changes in the production of TRP observed in CSF relate with TRP levels in the blood. Interestingly, the concentration of serum TRP was significantly decreased from day 0 to day 4 following TBI with a reduction ranging between 25 and 50 %, *p* < 0.05 (Fig. 3). Although the medians of TRP on day 5 (36.29 μM, 25–75 %: 29.34–44.37 μM) and day 4 (35.6 μM, 25–75 %: 30.63–43.93 μM) were similar, the apparent increase of TRP on day 5 was not found to be statistically different to control levels (*p* = 0.274), possibly due to a larger data variance at this time point.

### Up regulation of IDO1 mRNA in the injured brain supports the increase of QUIN in CSF

This section of the study aimed at elucidating the mechanisms leading to the alterations in KP metabolite detected in the CSF of TBI patients and substantiating the evidence of KP activation directly on the injured brain. Using post-mortem brain tissue from TBI victims, we examined changes in the expression level of six enzymes of the KP, namely IDO1, KYNase, KAT-II, KMO, 3HAO and QPRTase, which were normalised to a set of three constitutive genes to generate reliable PCR data (see the ‘Materials and methods’ section).

**Fig. 4:**
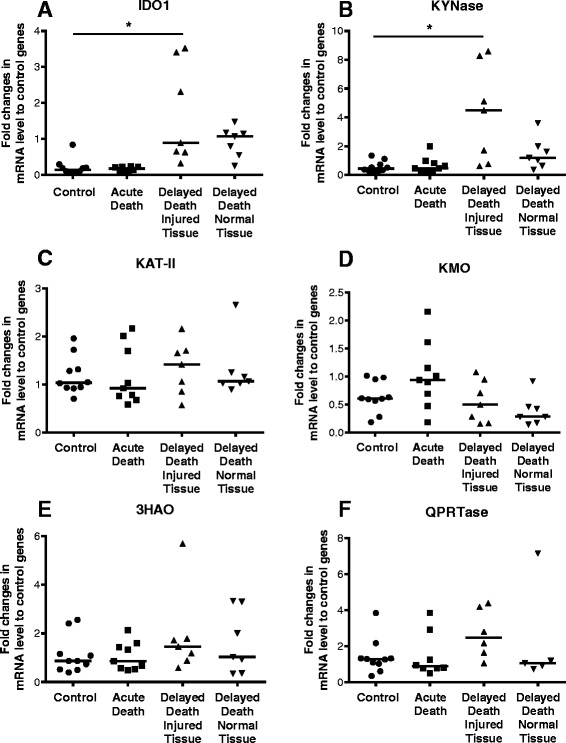
qPCR analysis of enzymes of the kynurenine pathway in brains of TBI victims demonstrates enhanced IDO1 and KYNase expression. Gene expression of six enzymes of the kynurenine pathway was determined by qPCR in four groups of post-mortem brains obtained from the temporal cortex after TBI and uninjured control individuals (*n* = 10). Groups comprise the following: TBI patients with acute death (survived <17 min after injury, *n* = 9), TBI patients with delayed death (survival between 6 and 122 h, *n* = 7) with tissue collected from brain regions having evident tissue pathology (Delay Death Injured Tissue) and brain regions without macroscopic damage obtained from the contralateral uninjured side (Delay Death Normal Tissue) (*n* = 7). **a** Indoleamine-pyrrole 2,3-dioxygenase (IDO1), **b**. kynurenase (KYNase), **c** kynurenine amino transferase-II (KAT-II), **d** kynurenine 3-monooxygenase (KMO), **e** 3-hydroxyanthranilic acid oxygenase (3HAO) and **f** quinolinic acid phosphoribosyl transferase (QPRTase). *Asterisks* indicate significant differences (*p* < 0.05) between TBI and control groups using one-way ANOVA followed by Dunnett’s multiple comparison as *post hoc*. Data shown as median with 25–75 percentile. IDO1 and KYNase were significantly increased in the Delay Death Injured Tissue group as to the control samples

IDO1 is the first enzyme required in the initial step of the KP to metabolise TRP into KYN. KYNase is needed for metabolising 3HK into 3HAA prior to the final conversion of the latter into QUIN. IDO1 mRNA levels were significantly increased at least fivefold in the Delayed Death Injured Tissue as compared to the controls (*p* < 0.05; Fig. 4a). There were no significant differences observed on the IDO1 levels between the other groups (i.e. Acute Death vs control and Delayed Death Normal Tissue vs control). A similar pattern was also observed in the KYNase mRNA levels whereby over a sevenfold increase was detected in the Delayed Death Injured Tissue over the controls (Fig. 4b, *p* < 0.05) and no differences between the other groups. In contrast, mRNA expression of KAT-II, KMO, 3HAO and QPRTase remained unchanged in both the acute and delayed death groups (Fig. 4c–f). Thus, the marked increase in QUIN production observed in CSF following TBI is supported by the upregulation of mRNA expression of those enzymes of the KP involved in the synthesis of QUIN, IDO1 and KYNase.

### Expression of active IDO1 protein is increased in the injured brain

We next seek to determine the localisation of active IDO1 enzyme on post-mortem brain tissue to be related to the findings of upregulated IDO1 mRNA shown above. Using an antibody that specifically recognises activated IDO1, immunohistochemistry revealed a significant increase in the number of IDO1-positive cells after TBI, which peaked in acute TBI and gradually declined in the injured and normal tissue of delayed death patients.

**Fig. 5:**
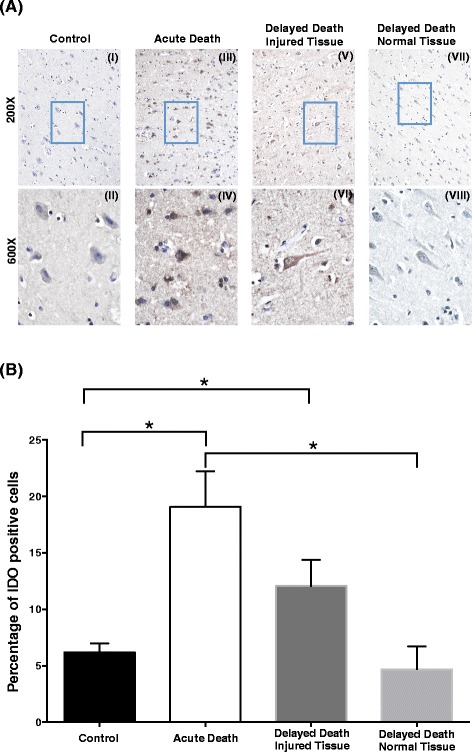
Immunohistochemistry of post-mortem brains reveals increased IDO1 acutely after TBI. **a** IDO1 immunohistochemistry was performed on post-mortem brains as described in the ‘Materials and methods’ section. IDO1 is found increasingly expressed in Acute Death and Delayed Death Injured Tissue samples compared to the control. Top panels (i, iii, v, vii) indicate ×200 magnification; bottom panels (ii, iv, vi, viii) indicate ×600 magnification. Figures represent IDO1 immunohistochemistry of the cortex of the uninjured control group (i and ii), acute death (<17 min after injury; Acute Death) (iii and iv), tissue obtained from injured cortex of TBI patients with delayed death (survival between 6 and 122 h; Delay Death Injured Tissue) (v and vi) and tissue collected from regions without macroscopic damage of the contralateral uninjured side (Delay Death Normal Tissue) (vii and viii). *Rectangular boxes* in the ×200 panels indicate the magnified regions of ×600. Brain sections were counterstained with haematoxylin. **b** IDO immunohistochemistry staining was semi-quantitatively assessed in post-mortem brain samples and presented as the percentage of positively stained cells relative to the total number of cells per field. Statistical differences were calculated as described in the Materials and methods section, *p ≤ 0.05. Data are mean ± SEM. Significant increase in the number of IDO1-positive cells was observed in Acute Death and Delayed Death Injured Tissue as compared to the controls; similar levels of IDO1-positive cells were between Control and Delayed Death Normal Tissue groups

The highest amounts of cells expressing IDO1 were detected in the brains of patients with acute death after TBI (19.1 ± 3.1 % of total cells; Fig. 5a(III,IV), Fig. 5b) compared to control tissues (6.2 ± 0.8 %; *p* = 0.0286; Fig. 5a(I,II)). Although the number of IDO1-positive cells was lower in the Delayed Death Injured Tissue, they were still significantly elevated (12.1 ± 2.3 %) compared to control (Fig. 5a(V,VI), Fig. 5b). For comparison, analysis of the brain region of the delayed death that did not present evident tissue pathology showed no increase in IDO1 protein expression, having similar numbers of IDO1-positive cells to control brains (Fig. 5a(VII,VIII), Fig. 5b). Based on the microscopic evaluation, cells expressing IDO1 have morphology typical of pyramidal cortical neurons, thus suggesting their potential contribution to the activation of the KP. Together, this immunohistochemical data supports the hypothesis that IDO1 is indeed activated in the injured brain.

### QUIN concentrations in CSF correlate with outcomes scores

**Fig. 6:**
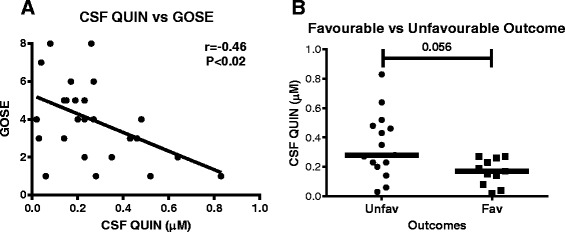
The concentration of QUIN in CSF is inversely correlated with GOSE scores and is significantly more elevated in patients with unfavourable outcome. **a** Correlative analysis was undertaken using maximal concentration of QUIN in CSF of each patient between day 0 (admission to hospital) and day 5 post-TBI and the GOSE scores assessed at 6 months after TBI. A significant negative correlation (*p* < 0.05) was observed between QUIN and GOSE scores with *r* = −0.46 (*n* = 28). **b** TBI patients were grouped into favourable (GOSE 5–8, *n* = 11) and unfavourable outcome (GOSE 1–4, *n* = 15) and their maximal QUIN concentration compared between the groups. Patients with unfavourable outcome showed a close to significant (*p* = 0.056) higher concentration of QUIN in CSF compared to patients with favourable outcome

Previous studies reported that higher concentrations of QUIN in CSF correlated with mortality in adult and paediatric TBI patients [28, 29]. We, therefore, investigated the relationship of QUIN with outcome scores at 6 months post-TBI. We performed a Pearson correlation of coefficient between the GOSE scores and maximal CSF concentration of QUIN between days 1 and 5 after TBI. A significant inverse correlation was observed between QUIN and GOSE with *r* = −0.46 and *p* < 0.02 (Fig. 6a). This correlation indicates a potential prognostic value for QUIN, whereby patients with higher QUIN production in the acute phase after injury have poor recovery at 6 months post-injury. Furthermore, we dichotomised TBI patients according to favourable (GOSE 5–8) and unfavourable outcome (GOSE 1–4) scores. Patients with unfavourable outcome had a close to significant higher concentration of QUIN (median: 0.28 μM, 25–75 %: 0.20–0.48 μM) than patients with favourable outcome (median: 0.17 μM, 25–75 %: 0.08–0.26 μM) (*p* = 0.056; Fig. 6b).

## Discussion

Clinical studies have shown that secondary injury processes induced by TBI generate ongoing disease-like characteristics that cause progressive brain damage, delay physical recovery and contribute to the onset of mental illness. Therefore, early treatment aimed to reduce acute pathological sequel is the major focus in the development of therapeutic strategies in TBI patients. It is well established that excitotoxicity, neuroinflammation and oxidative stress are pivotal pathways of secondary brain damage following TBI [38]. Studies of neurodegenerative diseases and infection of the nervous system have shown that TRP metabolism participates in the regulation of immune activation, generation of oxidative radicals and production of excitotoxic substances [17]. Despite robust evidence showing that the KP plays an important role in neuropathology, this pathway has only been sporadically examined in the context of TBI. This gap spurred the current investigation aimed at measuring a number of metabolites of the KP in live, severely injured TBI patients’ CSF and understanding how these metabolites relate to the expression of critical enzymes of the KP in post-mortem brain tissues of TBI victims. Our data demonstrates that the KP is profoundly activated after brain trauma by showing increased concentration of a number of TRP metabolites in CSF, including KYN, KYNA and QUIN. Enhanced production of the excitotoxic metabolite QUIN likely arises from the activation and overexpression of the enzyme IDO1 subsequently driving the pathway into the production of intermediate metabolites 3HK (passive intermediate) and 3HAA via the activation of KYNase.

We need to distinguish between IDO1 enzyme activation detected by immunohistochemistry and upregulation of IDO1 mRNA measured by PCR to explain the different timing of the changes observed. IDO1 is stored in a dephosphorylated form (inactive) with its activation by phosphorylation occurring within a few minutes from stimulation [39, 40]. This activation process makes IDO1 recognisable to the mAb used in immunohistochemistry of post-mortem early-injured brains presented in Fig. 5. Unfortunately, there is no commercially available antibody for the dephosphorylated/inactive form of IDO1 to allow for its detection *in situ*. Moreover, activated IDO1 has the capacity of auto-amplification, a fact that may explain the later upregulation of IDO1 mRNA in post-mortem brains of delayed survival times between 6 and 122 h post-TBI [39] (Fig. 4a).

In regard to the immunohistochemistry experiments, our data has to be taken with caution given the limited number of tissues stained and the variety of brain regions analysed. Importantly, however, these experiments confirm that active IDO1 protein is indeed present in the brain early after trauma, corroborating the hypothesis that IDO1 activation is actually increased within a few minutes after TBI (Fig. 5a,b). We are aware that these results warrant further investigations to explore the mechanisms and profiles of IDO1 activation after TBI to allow for a comprehensive quantification and understanding of its role in the ultimate activation of the KP.

Collectively, the analysis of the KP metabolite in CSF and the expression of the KP enzymes in post-mortem brain tissue suggests that the QUIN production is increased via the activation of the minor branch of the KP, beginning from the metabolism of KYN to AA, the production of 3HAA and finally the accumulation of QUIN. This hypothesis is also supported by the gradual increase in the concentration of AA in CSF (Fig. 1e), which, although not significant, displays a pattern strikingly similar to the pattern observed for QUIN production (Fig. 1d).

In addition, we report of an inverse correlation between elevated QUIN in CSF and the adverse long-term outcome, corroborating both a detrimental role of QUIN in the pathophysiology of secondary brain damage as well as the potential therapeutic implications of targeting QUIN production to reduce morbidity in this patient population.

The KP represents the major metabolic cascade of TRP metabolism, which degrades up to 90 % of available amino acid. The KP is dramatically enhanced in disease conditions that particularly involve the activation of the immune system. We and others have focused much of our research efforts in characterising the inflammatory response resulting from TBI, via clinical and experimental investigations (reviews [41, 42]).

During an inflammatory response, the release of cytokines, in particular IFN-γ, by activated monocytes and leukocytes increases the degradation of TRP via the KP [43]. This likely occurs through the ability of IFN-γ to activate the upstream enzyme IDO1 of the KP, which may become highly processed to release bioproducts such as QUIN, known to promoting excitotoxicity. We have previously shown that IFN-γ is rapidly upregulated in the CSF of severe TBI patients. In addition, using the same brain homogenates analysed in this study, we demonstrated that IFN-γ was already found enhanced in early death brains with even greater concentrations in the delayed death brains by a sixfold increase from controls [36, 44]. In fact, among the eight cytokines analysed, IFN-γ was the third highest cytokine following IL-6 and IL-8 [36].

We cannot exclude, however, the contribution of other factors in the activation/upregulation of IDO1. In fact, *in vitro* studies on cultured microglia have demonstrated IFN-γ-independent activation of IDO1 by TNF-α and IL-1β [45]. This point is pertinent to our study as we have previously shown a fourfold elevation of TNF-α at protein/mRNA levels in the same post-mortem brains harvested within 17 min post-injury while a nonsignificant increase of IL-1β mRNA was observed early (17 min) which was followed by a substantial and significant upregulation of over fivefold in the delayed group dying beyond 6 h from TBI [36]. It is conceivable that combined increase of these cytokines early after TBI results in subsequent and significant induction of IDO1 mRNA that was observed in the injured brain of delayed death patients (Fig. 4a).

In our study, the levels of TRP in CSF remained within a normal range whereas the blood concentrations were found below physiological values during almost the entire acute phase post-TBI. It is important to note that in healthy conditions the concentration of TRP in serum is tenfold higher than the CSF level. Although serum TRP decreased up to 50 % after injury, its blood levels were still higher compared to CSF. The striking difference between these two compartments may allow sufficient amounts of TRP to be transported to the brain or CSF via the brain/CSF barrier to maintaining a constant CSF concentration, which may explain the lack of changes of TRP in CSF.

Decreased concentrations of TRP in serum were reported previously in patients with infectious and neurodegenerative diseases and cancers. The authors speculated that this phenomenon is caused by an enhancement in peripheral IDO1/TDO enzyme activity [46]. Our observation of a significant decrease of TRP in serum after TBI confirms the findings reported by others in multiple trauma patients showing that blood TRP was significantly attenuated [46].

Although cytokines can strongly affect the KP, it has been demonstrated that TRP itself possesses important immune-regulatory properties. TRP depletion suppresses the proliferation of mononuclear cells [5], decreases immune cell activation [6, 47] and inhibits parasitic growth [48]. Therefore, a low concentration of serum TRP may provide some beneficial effects in the early stage after severe brain injury by reducing acute inflammatory responses. However, with the current setting, our study is unable elucidate the underlining mechanisms leading to the reduction of TRP in blood, a question which warrants further investigations.

Analysis of gene expression of the KP enzymes indicated that KP is strongly activated after TBI. The enzyme KAT has four isoforms, which are responsible for the synthesis of the neuroprotectant KYNA from its precursor KYN [49]. In this study, we have only examined KAT-II, one of the major KATs involved in both normal and disease conditions, which is considered as a promising target for pharmaceutical intervention. The expression of KAT-II in post-mortem brain did not vary between the three groups of TBI patients, Acute Death, Delayed Death Injured Tissue and Delayed Death Normal Tissue relative to control tissues. The unchanged expression levels of KAT-II observed in these brains are consistent with the unaltered concentration of its bioproduct KYNA in CSF between days 0, 1 and 2 and controls (Fig. 1c). It is important to consider that the average survival time of the delayed group is 40 h; thus, the tissue sample collected from these victims occurred well before the observed increase of KYNA in CSF, which was only found to be significant at 3 days post-injury. Here, we cannot adequately explain the mechanisms leading to enhanced KYNA and whether this is due to the increase of KAT expression/activation or as a result of an elevated concentration of the precursor KYN. However, we have to bear in mind that mRNA expression levels do not reflect changes in enzyme activation, thus posing a limitation in this study, solely based on qPCR. Previous work by Han et al. has shown that the Km of KAT-II is 1.7 ± 0.5 mM [50], a concentration that greatly exceeds the concentration of KYN in the CSF samples we found in the control and TBI cases (mean 0.08 ± 0.01 μM and 0.21 ± 0.02 μM, respectively). Therefore, we cannot state without doubt that the marked increase in the KYNA on days 3, 4 and 5 is attributed to the elevation of KYN following injury.

KMO is the key enzyme converting KYN into 3HK and subsequently into QUIN. This step of the KP has been suggested to be the main pathway for QUIN production. Inhibition of KMO was effectively ameliorating QUIN-mediated neurodegeneration in rodent [51]. However, in our study, KMO did not change significantly after TBI with median levels remaining similar among the four groups examined (Fig. 4d).

Newly emerged evidence demonstrated the important roles of 3HAA and AA in both brain and periphery in normal and disease conditions (see review by Darlington et al. 2010 [52]). Studies on osteoporosis [53], Huntington’s disease [54, 55], stroke [32] and depression [56] have shown a simultaneous increase in AA and reduction in 3HAA in blood. In the brain, AA is an effective inhibitor of 3HAO [57]; therefore, the increase in AA in neurodegenerative diseases may provide some degree of inhibition of 3HAO that is required for the synthesis of the neurotoxin QUIN. Our study demonstrated that the ratios of 3HAA and AA are higher in the early days after TBI and gradually decreased, suggesting an overproduction in AA compared to 3HAA over time.

Despite the lack of changes in those metabolites upstream to QUIN synthesis (3HAA, 3HK not measured) and despite the elevation of KYNA—a metabolite generated via a separate branch from the one responsible for QUIN production—evidence for enhanced activation of the branch of the KP pathway leading to increased QUIN reported in this study is based on the following data: (1) prolonged elevation of QUIN in CSF of TBI patients, (2) increased QUIN:KYN and QUIN:KYNA ratios from CSF measurements of these metabolites, (3) increased IDO1 and KYNase mRNA expression in human brains and (4) enhanced acute expression of IDO1 protein on neurons in the injured brain region but not in tissue areas of normal appearance.

QUIN exerts its neurotoxic activity by interacting with a subgroup of NMDA receptors leading to a widespread destruction of neuronal terminals and sparing fibres [58]. In cultured cortico-striatal neurons, prolonged exposure to submicromolar concentrations of QUIN caused excitotoxic damage [59]. Further, *in vivo* studies using intrastriatal injection of QUIN showed tissue lesions similarly located in the basal nuclei as occurring in Huntington’s disease patients [60]. Another recent *in vivo* study also demonstrated that brain regions, i.e. cerebellar cortex, hippocampus and cerebellum, have different susceptibility to QUIN-induced oxidative stress [61].

Our recent studies in NSC-34, a mouse motor neuron cell line, demonstrated that 2 μM QUIN is highly toxic and that uptake of QUIN by NSC-34 cells occurs as early as 30 min after exposure to QUIN [62]. QUIN neurotoxicity is mediated by both direct and indirect mechanisms. It has been reported that QUIN can directly increase free radical generations once entering the cells [63–65]. Free radical production following hypoxia-ischemia in association with inflammation plays a prominent role in the ultimate death of cells. Free radicals can cause cellular injury via several mechanisms, including membrane lipid peroxidation, oxidative damage of proteins and DNA and RNA fragmentation [66]. QUIN has been shown to chelate Fe^2+^ to form a QUIN-Fe^2+^ complex, which results in the production of the most toxic hydroxyl radical via the Fenton reaction [67]. Accumulation of QUIN in neurons has been shown in Alzheimer’s disease [9], motor neurons in ALS [68] and neuronal cell line [62]. Collectively, these multiple biochemical pathways in which QUIN may be involved have been demonstrated to be contributing elements within the complex mechanisms of secondary brain damage arising from TBI.

Studies have shown significant associations between QUIN concentration and severity of the infectious diseases of the nervous system. Concentration of CSF QUIN is elevated 3.5-fold in the early stage of immunodeficiency syndrome (AIDS) patients [18], while it increased over 20-fold in patients at the late stage of the disease [18]. A higher level of CSF QUIN in individuals with HIV infection was positively correlated with more severe motor deficits [69]. In the context of outcome following TBI, these observations are consistent with the finding of our study, whereby we detected a significant negative correlation between patients’ GOSE scores and QUIN concentrations in CSF, including the evidence of higher QUIN in patients with unfavourable when compared to those with favourable outcomes. This suggests a critical role of QUIN as a potential biomarker for the early prediction of long-term outcome following TBI.

## Conclusion

Highly neuroactive products of TRP metabolism have received considerable attention in the last two decades. Numerous studies have demonstrated the importance of TRP and its metabolites in both normal physiology and disease conditions such as neuroinflammatory, infectious and neurodegenerative diseases. However, limited reports have examined changes and roles of TRP metabolism in TBI. This study is the first to investigate longitudinal changes in key metabolites of the KP in patients with severe TBI including investigations in brain gene expression of key regulatory enzymes of the KP in individuals who died due to TBI. We have obtained novel and convincing data of enhanced KYNase expression occurring in the acute phase following TBI. These results indicate that TBI may selectively activate the branch of the KP leading to increased QUIN production in the injured brain. This novel finding supports the concept that blocking the activity of KYNase (with existing compounds such as oestrone sulphate or nicotinylalanine) may become an effective therapeutic strategy in attenuating QUIN production and its driven excitotoxicity, thus diminishing the detrimental consequences of secondary brain damage.
